# An exploration of elevated HIV and STI risk among male sex workers from India

**DOI:** 10.1186/1471-2458-13-1059

**Published:** 2013-11-09

**Authors:** Prakash Narayanan, Anjana Das, Guy Morineau, Parimi Prabhakar, Gururaj Rao Deshpande, Raman Gangakhedkar, Arun Risbud

**Affiliations:** 1 FHI 360, India Country Office, New Delhi, India; 2 FHI 360, Asia Pacific Regional Office, Bangkok, Thailand; 3 India HIV/AIDS Alliance, Hyderabad, India; 4 National AIDS Research Institute, Pune, India

**Keywords:** Male sex worker, Men who have sex with men, HIV, India

## Abstract

**Background:**

Men who have sex with men (MSM) who also report transactional sex (male sex workers or MSWs) are known to be at higher risk for HIV and sexually transmitted infections (STIs). The study aimed to profile socio-demographic characteristics and risk factors associated with high HIV prevalence among MSWs.

**Methods:**

A cross-sectional study was conducted in 2008–9 among 483 high-risk MSM who attended STI clinics at Mumbai and Hyderabad, two large cities in India.

**Results:**

About 70% of the MSM reported transactional sex. As compared to other MSM, MSWs had more male partners (8.9 versus 2.5, p < 0.001) and higher rates of receptive anal sex (96% versus 72%, p < 0.001). HIV prevalence among MSWs and other MSM was 43.6% and 18.1% respectively. HIV prevalence among MSWs was associated with the place of residence (MSWs from Hyderabad were 7.3 times more likely to be infected), positive syphilis serology (3.8 times) and duration of sex work (increased by 8% for every additional year).

**Conclusion:**

The study showed that MSWs are at high risk for HIV acquisition/transmission, which highlights the need for intensified interventions for personalized risk-reduction counselling and STI screening. Newer biomedical interventions such as pre-exposure prophylaxis and treatment as prevention could also be considered.

## Background

In the first decade of 2000, HIV prevalence among men who have sex with men (MSM) increased in many countries across the world [1-5]. HIV prevalence estimates computed using pooled data from seven Asian countries (Thailand, Vietnam, Cambodia, China, Indonesia, India and Nepal), found that MSM are 19 times more likely to get infected than other men of reproductive age [1]. MSM are particularly vulnerable to HIV because of their wide range of HIV-related risk behaviours including high number of sexual partners, bisexual behaviour, inconsistent condom use, substance use, transactional sex, and low levels of awareness on HIV and sexually transmitted infections (STIs) [6,7]. Studies conducted among MSM in low and middle-income countries have demonstrated that HIV risks are associated with a variety of sexual partnerships and sexual practices [2,8]. In India, MSM who sell sex (male sex workers or MSWs) have a greater risk for HIV and STIs than other MSM [9-11]. MSWs are more vulnerable to HIV than other MSM because MSWs need to fulfil the desires of their clients and, in some instances, agree to unprotected sex, particularly when there is a scarcity of clients [12]. While MSWs may serve as HIV core transmitters to their clients, they may also function as a bridge group in the transmission of HIV and other STIs to the general population because they also engage in non-commercial sex with male and female partners [13].

Even though male to male sex has recently been decriminalized in India, social stigma may hinder risk-reduction and access to HIV prevention interventions [14]. Programme data from 2006 estimated that there were 20,800 high-risk MSM in Mumbai and 5,000 in Hyderabad [15]. Using the proportion of self-reported transactional sex in the 2007 surveillance survey among MSM, the numbers of MSWs are estimated to be 7,700 in Mumbai and 1,800 in Hyderabad [16].

The Indian National AIDS Control Programme has recognised the importance of HIV prevention for MSM and supports NGOs providing targeted interventions for high-risk MSM (HR-MSM), defined as MSM with multiple sexual partners [17]. However, the interventions are uniform for MSM, male-to-female transgendered persons (TGs) and MSWs [17]. The prevention package includes peer-led outreach education, promotion and distribution of condoms and lubricants, STI clinical services, community mobilization and structural interventions. STI clinics provide services for management of STI syndromes, periodic STI check-ups and syphilis screening. Additionally, HIV voluntary counselling and testing (VCT) services are provided either on-site or through referrals.

This study presents the socio-demographic characteristics and HIV/STI prevalence of MSWs. The authors investigate the factors associated with HIV infection among MSWs and highlight the need for tailored services for this population.

## Methods

A cross-sectional study was conducted during 2008–2009 in four dedicated MSM clinics, run by NGOs implementing HIV prevention programmes for HR-MSM and TGs. The clinics were located in Mumbai and Hyderabad, two large cities in India. Eligible participants were MSM clinic attendees aged 18 years or more, who reported having had sex with at least two male partners in the last month and were not under the influence of alcohol or drugs at the time of consultation. MSM were mobilized to participate in the study by peer educators through the outreach. However, all clients attending the clinics during the study period were screened for eligibility. Informed consent was administered to eligible MSM. Participants received a compensation corresponding to the cost of transport to the clinic. The study protocol was approved by the institutional ethical review committees of the National AIDS Research Institute (NARI) of India and the FHI 360 Protection of Human Subjects Committee in the USA. All staff was trained on study procedures.

### Data collection

Participants were interviewed by male investigators who collected information on demographic characteristics, sexual practices, condom use and previous exposure to HIV prevention interventions. Physicians enquired about symptoms of STIs, examined the participants and provided free treatment as per the national guidelines for STI management. The physicians collected rectal swabs during clinical examination. Participants were asked to provide first void urine and a laboratory technician collected 5 ml of whole blood through venepuncture.

### Laboratory investigations

All specimens were stored at -20°C and shipped for testing at NARI in Pune, India. Rectal swabs were tested for *Neisseria gonorrhoeae* (NG) and *Chlamydia trachomatis* (CT) by polymerase chain reaction using Roche Amplicor (Roche Molecular Diagnostics, CA, USA). Urine specimens were tested for NG and CT using APTIMA Combo-2 Assay (Gen-Probe Inc., San Diego, USA). Sera were tested for HIV using a three tests sequential algorithm including Microlisa-HIV (ELISA test, J Mitra & Company Private Limited, New Delhi, India ) for screening and two rapid tests conducted in parallel for confirmation: HIV Tridot (J Mitra & Company Private Limited, New Delhi, India), and CombiAids (Span Diagnostics Ltd, Surat, India). The HIV sero-status of the sera with discrepant results was determined by Western blot (Genetic Systems HIV-1 Western Blot, Bio-Rad laboratories, Redmond, WA, USA). Syphilis screening was performed using rapid plasma reagin test (RPR, Span Diagnostics Ltd, India); reactive sera was confirmed by *Treponema pallidum* hemagglutination assay (TPHA) using Syphagen-TPHA (Biokit, Barcelona, Spain). Hence positive syphilis serologies include both active syphilis and serologic scars. Herpes simplex virus type 2 (HSV-2) antibodies (IgG) were detected with ELISA using EIA-Herpeselect-2 IgG kit (Focus Diagnostics Inc, Cypress, California USA).

### Statistical analysis

Data was double entered and compared using CSPro (US Census Bureau) and analysed using STATA 12.1 (StataCorp LP, College Station, Texas, USA). Those self-identifying as TGs were excluded from the present analysis because their sexual behaviour differs from MSM.

MSWs were defined as those who had ever received cash or kind in exchange for sex. The analysis compares the socio-demographic, behavioural characteristics and STI/HIV prevalence between MSWs and other MSM (who did not report transactional sex). Statistical differences between categorical variables were assessed using the Chi-square or Fisher exact test (if cell value <5) and means were compared using the Student’s T-test. Duration of selling sex was calculated as the difference between current age and reported age at first commercial sex. Duration of sex work was used to categorize MSWs into four groups: 0–1 year, 2–5 years, 6–10 years and 11–38 years. Respondents’ reported characteristics and prevalence of STIs and HIV were compared among the four groups. Differences were tested using the Pearson Chi^2^ test and trends were assessed using a Chi^2^ for trends (Chi^2^-trends). Means were compared using the Kruskal-Wallis test that addresses the absence of normal distribution in groups with a small number of observations.

Finally we conducted logistic regression to assess the factors associated with HIV and HSV-2 among MSWs. All variables associated with HIV with a level of significance p < 0.2 in bivariable analysis were included in a multiple logistic regression model. The final model was obtained using backward stepwise elimination. All tests were double sided and a p-value smaller than 0.05 was considered statistically significant. Some of the p-values for Chi^2^ were not reported because of insufficient cell size (n < 2).

## Results

A total of 551 eligible MSM were contacted for the study, of them 22 refused participation. Of the 529 enrolled study participants, 17 had incomplete data (either behavioural or biological). Among the 512 MSM with complete data, 29 TGs were excluded from the present analysis. The 483 MSM retained for analysis included 326 (67%) from Hyderabad and 157 (33%) from Mumbai.

Participants’ mean age was 26.8 years (SD = 5.9). About one third (30%) were currently married and 39% were living with sexual partners; 80% were literate and 69% reported current use of alcohol/drugs (Table 1). A majority of MSM (57%) self-identified as *kothis*: i.e., reporting a receptive anal sex-role. Almost half (42%) reported having had sex with women in the past three months. About one-fifth (19%) acknowledged having ever paid for sex. Condom use at last sex was relatively high for receptive anal sex (89%) with a male partner, but decreased for insertive anal sex with males and females to 79% and 39% respectively. Consistent use of condoms with non-commercial partners was very low (47% with male partners and 8% with female partners).

**Table 1:** Demographics and sexual practices of MSW and other MSM

**Background characteristics**	**Ever sold sex (MSW)**	**Never sold sex (Other MSM)**	**p-value**	**Total**
	**% (n = 334)**	**% (n = 149)**		**% (n = 483)**
**Place of study**				
Hyderabad, Andhra Pradesh	82.0 (274)	34.9 (52)	<0.001	67.5 (326)
Mumbai, Maharashtra	18.0 (60)	65.1 (97)		32.5 (157)
**Demographics**				
Age <25 years	41.0 (137)	46.3 (69)	0.278	42.7 (206)
Currently married	31.7 (106)	27.5 (41)	0.352	30.4 (147)
Literate	77.0 (257)	87.3 (130)	0.009	80.1 (387)
Living with sexual partner (male or female)	43.1 (144)	30.9 (46)	0.011	39.3 (190)
Self-reported typology				
*Kothis* – receptors or bottoms	65.6 (219)	37.6 (56)	<0.001	56.9 (275)
*Panthis* – inserters or tops	3.0 (10)	20.1 (30)		8.3 (40)
*Double-deckers* – both receptive & insertive	31.4 (105)	42.3 (63)		34.8 (168)
**Sexual partners**				
Sex with women in the past 3 months	39.5 (132)	48.3 (72)	0.070	42.2 (204)
Ever paid for sex	19.2 (64)	18.1 (27)	0.787	18.8 (91)
Commercial sex with women (sold or bought) in past 7 days	5.4 (18)	2.0 (3)	0.145	4.4 (21)
Sold sex in past 7 days	94.6 (316)			
Paid other men for having sex in past 7 days	13.2 (44)	11.4 (17)	0.590	12.6 (61)
Mean number of male partners in the past 7 days (SD)	8.9 (8.6)	2.5 (2.9)	<0.001	7.0 (7.9)
**Sexual practices reported for the past 3 months**				
Receptive anal sex	96.1 (321)	71.8 (107)	<0.001	88.6 (428)
Insertive anal sex with a male partner	38.3 (128)	54.4 (81)	0.001	43.3 (209)
Insertive vaginal sex	37.1 (124)	45.0 (67)	0.104	39.5 (191)
Insertive anal sex with a female partner	6.6 (22)	10.1 (15)	0.184	7.7 (37)
**Condom use**				
At last receptive anal sex with male partner	87.9 (282)	67.3 (72)	<0.001	82.7 (354)
At last insertive anal sex with male partner	82.0 (105)	74.1 (60)	0.169	79.0 (165)
At last insertive vaginal sex	41.1 (51)	50.8 (34)	0.202	44.5 (85)
At last insertive anal sex with female partner	45.5 (10)	26.7 (4)	0.314	37.8 (14)
Consistent condom use with non-commercial male partner	49.8 (113)	41.7 (53)	0.146	46.9 (166)
Consistent condom use with non-commercial female partner	5.3 (12)	11.8 (15)	0.027	7.6 (27)
**Other risk factors, knowledge and uptake of STI services**				
Current use of alcohol or drugs	74.3 (248)	55.7 (83)	<0.001	68.5 (331)
Heard about STIs	73.4 (245)	68.5 (102)	0.269	71.8 (347)
Self-perceived as at high risk for STI acquisition	16.2 (54)	8.7 (13)	0.029	13.9 (67)
Ever visited the intervention clinic	75.2 (251)	83.9 (125)	0.033	77.9 (376)

### Characteristics of the men who sell sex

Two-thirds (69%) of the MSM had ever sold sex to male clients, though only 19% reported sex work as the primary source of income. The proportion of MSWs in the total MSM population was higher in Hyderabad as compared to Mumbai (84% versus 38%). Of those who had ever sold sex, 95% had done so in the past seven days. The median and mean duration of selling sex were 5 years and 6.7 years (SD = 5.3), respectively.

Compared to MSM who did not report selling sex (Table 1), MSWs were more likely to be living with a sexual partner (43% versus 31%, p = 0.011); a higher proportion self-identified as anal-receptive/*kothis* (66% versus 38%, p < 0.001) and reported receptive anal sex in the past three months (96% versus 72%, p < 0.001); and a smaller proportion reported insertive anal sex with male partners (38% versus 54%, p = 0.001). In addition, MSWs reported a higher number of male partners in the past seven days than other MSM (mean 8.9 versus 2.5, p < 0.001). Compared to other MSM, MSWs reported higher condom use at last receptive anal sex (88% versus 67%, p < 0.001) and lower consistent condom use with non-commercial female partners (5% versus 12%, p = 0.027). However, differences between MSWs and other MSM were not statistically significant for condom use at last insertive sex with either male or female partners. More MSWs than other MSM reported currently using drugs or alcohol (74% versus 56%, p < 0.001). Self- perception of risk for acquiring STIs was higher among MSWs (19% versus 9%, p = 0.029). Compared to other MSM, MSWs were more likely to have received free condoms through the outreach (91% versus 81%, p = 0.001). However, a lower proportion of MSWs had ever attended the STI clinic (75% versus 84%, p = 0.033). MSWs who had ever visited the clinic prior to the survey reported higher levels of consistent condom use with clients (62% versus 45%, p = 0.005).

### Prevalence of sexually transmitted infections and HIV

Results of the laboratory testing for STIs and HIV are presented in Table 2. The prevalence of curable STIs (including syphilis, rectal CT and/or NG, and urethral CT and/or NG) among all participants was 21.7%, including 6.6% syphilis, 11.4% rectal NG, 4.8% rectal CT, and 2.1% urethral CT and/or NG. There was no statistical difference in the prevalence of curable STIs among MSWs and other MSM. The most common infections among all participants were HSV-2 (50.5%) and HIV (35.6%). The prevalence of these synergistic infections was higher among MSWs than other MSM (HIV 43.6% versus 18.1%, p < 0.001, and HSV-2 59.9% versus 29.5%, p < 0.001).

**Table 2:** Prevalence of STIs and HIV among MSWs and other MSM

**Prevalence of STIs**	**MSW**	**Other MSM**	**p-value**	**Total**
	**% (n = 334)**	**% (n = 149)**		**% (n = 483)**
Syphilis serology (RPR + TPHA)	7.8 (26)	4.0 (6)	0.125	6.6 (32)
Urethral NG/CT	3.0 (10)	0.0 (0)	NR^*^	2.1 (10)
Rectal CT	4.8 (16)	4.7 (7)	0.965	4.8 (23)
Rectal NG	9.9 (33)	14.8 (22)	0.119	11.4 (55)
Rectal NG/CT	13.5 (45)	18.1 (27)	0.185	14.9 (72)
Any curable STI (syphilis, Rectal NG/CT, Urethral NG/CT)	21.6 (72)	22.2 (33)	0.884	21.7 (105)
HSV-2	59.9 (200)	29.5 (44)	<0.001	50.5 (244)
HIV positivity	43.6 (143)	18.1 (27)	<0.001	35.6 (170)

*Not Reported because of insufficient cell size (n < 2).

HSV-2 and HIV prevalence varied significantly with the reported status of transactional sex; HSV-2 and HIV prevalence were 61.4% and 44.8% among those who reported selling sex in the last seven days, 33.3% and 22.2% among those who had ever sold sex but not in the last seven days, and 29.5% and 18.1% among those who had never sold sex.

### Characteristics of MSWs by city

Prevalence of HIV among MSWs differed by place of residence: 10.5% in Mumbai and 50.6% in Hyderabad (p < 0.001). Compared with Mumbai, MSWs from Hyderabad were older (mean age 24.1 years versus 27.5 years, p < 0.001); a higher proportion were currently married (9% versus 36%, p < 0.001), had sold sex in the past week (86% versus 96%, p = 0.005), reported receptive anal sex and vaginal sex in the last three months (90% versus 97%, p = 0.005, 25% versus 39%, p = 0.038), and used alcohol/drugs (51% versus 79%, p < 0.001). MSWs from Hyderabad also had a higher prevalence of HSV-2 (67.2% versus 22.8%, p < 0.001), rectal CT and/or NG (15.5% versus 5.3%, p = 0.054) and syphilis (8.5% versus 0.0%*).*

### Trends in risk factors by duration of selling sex among MSWs

We investigated the trends in demographics and behaviours among MSWs by duration of selling sex (Table 3). There were more literate MSWs among those new to the sex trade than those who had sold sex for longer periods (from 88% in the first year to 66% among those selling sex for more than 10 years; Chi^2^-trends p < 0.001). With an increasing duration in sex work, the mean age of MSWs increased (Chi^2^-trends p < 0.001) and an increasing proportion reported being currently married (Chi^2^-trends p = 0.004) or living with a sexual partner (Chi^2^-trends p = 0.041). Among those with a longer duration in sex work, the proportion self-identifying as anal receptive/*kothis* increased (43% in the first year and 79% after more than 10 years of selling sex; Chi^2^-trends p < 0.001), while the proportion who reported insertive anal sex with men in the past three months decreased (53% in the first year and 26% after more than 10 years of selling sex; Chi^2^-trends p = 0.003). MSWs who had been selling sex for more than 10 years reported a higher number of male partners in the last week (mean 10.6 partners) than those selling sex for a year or less (7.7 partners; Chi^2^-trends p < 0.001). The proportion reporting vaginal sex in the past three months increased from 28% in the first year to 48% after more than 10 years selling sex; Chi^2^-trends p = 0.049, whereas there was no statistically significant change regarding the proportion who reported having had sex with women in the past three months (p = 0.250). There were no statistical associations between condom use and duration of selling sex. Duration of selling sex was strongly associated with the HIV prevalence which increased from 33% in the first year to 57% among those selling sex for more than 10 years (Chi^2^-trends p = 0.001); and with HSV-2 prevalence which increased from 35% in the first year to 85% among those selling sex for more than 10 years (Chi^2^-trends p < 0.001). Self-perceived risk for acquisition of STIs increased from 8% in the first year to 29% after more than 10 years of selling sex (Chi^2^-trends p = 0.002). A longer duration of selling sex was associated with an increasing proportion of MSWs reporting current use of drugs or alcohol (from 65% in the first year to 77% after more than 10 years; Chi^2^-trends p = 0.012).

**Table 3:** Demographics and sexual practices of MSWs by duration of selling sex to men

**Characteristics**	**Duration selling sex (Years)**				**Chi**^**2 **^**p-value for trends**	**Total**
	**0-1**	**2-5**	**6-10**	**11-38**		
	**% (n = 40)**	**% (n = 124)**	**% (n = 97)**	**% (n = 65)**		
**Demographics**						
Mean age (SD)	22.6 (4.2)	24.7 (4.5)	27.5 (4.2)	33.1 (5.7)	<0.001	26.9 (5.8)
Literate	87.5 (35)	85.5 (106)	71.1 (69)	66.2 (43)	<0.001	77.6 (253)
Currently married	25.0 (10)	25.8 (32)	30.9 (30)	47.7 (31)	0.004	31.6 (103)
Living with sexual partner (male or female)	37.5 (15)	38.7 (48)	41.2 (40)	55.4 (36)	0.041	42.6 (139)
Self-reported receptors (*kothis*)	42.5 (17)	61.3 (76)	72.2 (70)	78.5 (51)	<0.001	65.6 (214)
**Sexual partners**						
Sex with women in the past 3 months	37.5 (15)	37.1 (46)	37.1 (36)	47.7 (31)	0.250	39.3 (128)
Sex with wife in past month	20.0 (8)	23.4 (29)	21.7 (21)	40.0 (26)	0.024	25.8 (84)
Mean number of male partners in the past 7 days (SD)	7.7 (6.6)	7.1 (7.6)	8.9 (10.2)	10.6 (11.5)	<0.001	8.6 (9.0)
Paid men for sex past 7 days	17.5 (7)	15.3 (19)	7.2 (7)	13.9 (9)	0.327	12.9 (42)
**Sexual practices reported for the past 3 months**						
Insertive anal sex with a male partner	52.5 (21)	41.9 (52)	34 (33)	26.2 (17)	0.003	37.7 (123)
Insertive vaginal sex	27.5 (11)	35.5 (44)	35.1 (34)	47.7 (31)	0.049	36.8 (120)
**Condom use**						
At last insertive anal sex with male partner	85.7 (18)	76.9 (40)	81.8 (27)	88.2 (15)	0.689	81.3 (100)
At last insertive vaginal sex	45.5 (5)	47.7 (21)	32.4 (11)	41.9 (13)	0.508	41.7 (50)
Consistent condom use with non-commercial male partner	35.7 (10)	50.6 (46)	48.3 (29)	57.1 (24)	0.162	49.3 (109)
Consistent condom use with non-commercial female partner	7.1 (2)	4.4 (4)	3.3 (2)	9.5 (4)	0.592	5.4 (12)
**Other risk factors, knowledge and uptake of STI services**						
Current use of alcohol or drugs	65.0 (26)	66.1 (82)	85.6 (83)	76.9 (50)	0.012	73.9 (241)
Heard about STIs	60.0 (24)	75.0 (93)	79.4 (77)	70.8 (46)	0.329	73.6 (240)
Self-perceived as at high risk for STI acquisition	7.5 (3)	12.9 (16)	15.5 (15)	29.2 (19)	0.002	16.3 (53)
**Prevalence of STIs and HIV**						
Syphilis serology	0.0 (0)	8.1 (10)	8.3 (8)	6.2 (4)	0.429	6.8 (22)
Rectal NG/CT	7.5 (3)	14.5 (18)	16.5 (16)	10.8 (7)	0.762	13.5 (44)
HSV-2	35.0 (14)	48.4 (60)	66.0 (64)	84.6 (55)	<0.001	59.2 (193)
HIV positivity	32.5 (13)	35.5 (44)	48.5 (47)	56.9 (37)	<0.001	43.3 (141)

### Correlates of HIV and HSV-2 positivity among MSWs

Table 4 shows the associated characteristics of MSWs who were HIV seropositive: those from Hyderabad as compared to those from Mumbai (p < 0.001), among older MSM (p < 0.001), current users of drugs or alcohol compared to non-users (p = 0.021), and those with positive syphilis serology compared to those with negative syphilis serology (p = 0.001). The risk of HIV infection increased 8% for each additional year in sex work. Prevalence of HIV was also higher among illiterate MSWs than those who were literate (p = 0.014), although one should note that literacy was a collinear of duration of selling sex that is associated with HIV prevalence. Four variables were retained in the multiple regression model. MSWs from Hyderabad were 7.3 times more likely to be infected with HIV than those from Mumbai (p < 0.001); MSWs with a positive syphilis serology were 3.8 times more likely to be infected with HIV (p = 0.002); those who had a positive HSV-2 serology were 12.9 times more likely to be infected with HIV (p < 0.001); and MSWs’ risk of getting infected with HIV increased 8% for every additional year selling sex (p = 0.002).

**Table 4:** Bivariable and multivariable analysis of factors associated with HIV and HSV-2 seropositivity among MSWs

	**HIV status**				**HSV-2 status**			
**Correlates**	**Bivariable**		**Multivariable**		**Bivariable**		**Multivariable**	
	**Odds ratio**	**95% CI**	**Odds ratio**	**95% CI**	**Odds ratio**	**95% CI**	**Odds ratio**	**95% CI**
Hyderabad city (ref Mumbai)	8.69^**^	3.61-20.92	7.32^**^	3.01-17.79	6.23^**^	3.30-11.79	4.00^**^	1.88-8.54
Illiterates	1.92^*^	1.13-3.24			1.93^*^	1.11-3.35		
Self-reported receptors – *kothis*	1.55	0.97-2.48			1.38	0.87-2.18		
Marital status (currently married)	1.59	0.99-2.55			2.42^**^	1.46-4.01		
Had sex with women in past 3 months	1.38	0.88-2.16			1.79^*^	1.13-2.84		
Consume alcohol/drugs	1.83^*^	1.08-3.08			1.96^*^	1.19-3.22		
Duration in sex work	1.08^**^	1.04-1.13	1.08^*^	1.03-1.13	1.17^**^	1.10-1.24	1.19^**^	1.11-1.27
Syphilis serology (RPR + TPHA)	5.18^**^	1.84-14.63	3.83^*^	1.36-10.79	2.37	0.93-6.07		
HSV-2 status	13.65^**^	7.50-24.88	12.92^**^	7.00-23.84				
HIV status					13.65^**^	7.50-24.88	10.70^**^	5.63-20.33

*p < 0.05 & p > 0.001; **p ≤ 0.001.

Bivariate analysis among MSWs found a higher prevalence of HSV-2 infection in Hyderabad than in Mumbai (p < 0.001), among older MSWs (p < 0.001),among illiterates as compared to literates (p = 0.020), among those currently married than those never married (p = 0.001), among those reporting sex with a female in the three months preceding the study than those with no female partners (p = 0.013), and among current users of drugs or alcohol compared to those with no recent intoxicant use (p = 0.008). Multivariate analysis showed that HSV-2 positivity among MSWs was 10.7 times more likely to be associated with a positive HIV sero-status (p < 0.001); four times higher among MSWs from Hyderabad than from Mumbai (p < 0.001); and the risk for HSV-2 infection increased 18% for every additional year selling sex (p < 0.001).

## Discussion

Our study showed that MSWs reported more HIV risk behaviours in comparison to other MSM, including a higher number of male sexual partners, a higher proportion reporting substance use and receptive anal sex, and a lower proportion accessing STI services. The prevalence of HIV and HSV-2 among MSM in general, and MSWs in particular, was high. HIV prevalence among MSWs was associated with the place of residence (higher in Hyderabad than Mumbai), HSV-2 and syphilis seropositive status and duration of sex work. Our survey also found a very high HIV prevalence of 57% among MSWs who had sold sex for more than 10 years. As the duration in sex work increased, MSWs were less likely to report insertive anal sex and more likely to self-identify as *kothis*/engaging in receptive anal sex. As compared to Mumbai, MSWs from Hyderabad were at higher risk for HIV possibly owing to several factors; they were older, a higher proportion were selling sex, reported higher rates of receptive anal sex and substance use, and had a higher prevalence of curable STIs.

The prevalence of HIV among MSM in our clinic-based study is substantially higher than the prevalence estimates obtained from a community-based study in 2009, the Integrated Behavioural and Biological Assessment, Round 2 (IBBA-2) that selected participants through time-location cluster sampling. However, the IBBA-2 also showed a higher HIV prevalence among MSM in Hyderabad than in Mumbai (25.8% and 3.8% respectively) [18]. Another study, conducted among MSM in the twin cities of Hyderabad and Secunderabad, which used a non-randomly generated sample from drop in centres and pubic cruising sites, reported an HIV prevalence of 22%; the multiple logistic regression analysis showed significant associations of seropositivity with age (>30 years), educational level (lower among post-graduates), history of unprotected anal intercourse and reported sexual positions (both unmarried and married receptive/*kothis* and unmarried dual/*double deckers*) [19]. Hyderabad and Mumbai are the capital cities of the states of Andhra Pradesh and Maharashtra respectively. The 2010–11 National HIV Sentinel Surveillance (HSS) showed that the HIV prevalence among pregnant women (used as a proxy for the general population) in the state of Andhra Pradesh is much higher than that of Maharashtra and the national average (Andhra Pradesh 0.76%, Maharashtra 0.42%, national average 0.40%) [4]. While the multivariate analysis for HIV seropositivity in our study included well-known individual risk factors, it is possible that the higher risk in Hyderabad may also be driven by other factors not measured such as a higher exposure to HIV through sexual networks of partners who have a higher HIV prevalence.

The prevalence of HSV-2 among MSWs (60%) is considerably higher than that among general male population in India (10%) [20]. As compared to MSM not selling sex, the high prevalence of HSV-2 among MSWs is consistent with their higher number of male partners, since HSV-2 in MSM is strongly associated with the number of lifetime sexual partners [21].

Reported consistent condom use was relatively low with non-commercial male partners and very low with female partners. MSM’s reported condom use at last sex was lowest for anal sex with female partners, which is consistent with studies showing that many MSM do not consider anal sex as a risk behaviour [22,23]. Almost half of the MSWs reported having sex with women and a small proportion also reported sex with female sex workers. In India, many MSM get married and engage in marital sex in response to familial and peer pressures, as well as social and cultural norms [24,25]. Our data also shows a rising trend of a 'currently married’ status among MSWs with an increasing duration in sex work. MSWs who have been selling sex for longer periods may play a substantial role in transmitting HIV to their wives and other female sexual partners because of high HIV prevalence and risky sexual practices.

Despite the fact that one out of four MSWs was infected with curable STIs, only a few perceived themselves at risk for STIs and a quarter had never visited the program clinic for STI services prior to the study. This gap in uptake of clinical services needs to be addressed. It is known that clinic visits provide opportunities for risk-reduction counselling, STI management, syphilis and HIV testing. A study among MSWs in Mumbai showed that HIV positives are 2.4 times more likely to be co-infected with life-time syphilis [11]. Our study also found a relatively high prevalence of syphilis among MSWs, which is alarming as syphilis was found to be a significant co-factor for HIV prevalence in our sample.

A majority of the MSM and more of the MSWs reported consuming alcohol or drugs. It has been demonstrated that substance use may be a mechanism used by MSM to cope with psychosocial pressures [26]. In Pakistan, for example, it is the MSWs’ daily preoccupation to conceal sex work activities and homosexuality from their families and a stigmatizing society [27]. Alcohol/drug use among MSM in India was found associated with high risk sexual behaviours, improper use of condoms and poor condom use negotiation [28].

There were several limitations in our study. Since the study was conducted in targeted intervention clinics, the participation of MSWs in our study could be disproportionately high and the prevalence of HIV/STIs and correlates may be different in MSM/MSWs who do not attend such facilities. Therefore, our results may not represent the general population of MSM/MSWs in the two cities. The small sample size of some subgroups/categories of MSWs may affect the statistical power of estimates and hence there is a need to interpret the data with caution. However, we have presented the analysis as there are limited studies on the risk characteristics of MSWs and they are an important group for HIV prevention. We did not collect data on prior HIV testing, which limits our understanding of MSWs possible uptake of treatment services. Finally, because this was a cross-sectional assessment, we cannot make causal inferences.

## Conclusion

Our study found that MSWs are extremely vulnerable to HIV and are likely to be core transmitters to their male and female partners, which highlights the need for intensified interventions for MSWs. Outreach workers should prioritize MSWs for prevention services through intensified risk-reduction education and promoting regular clinic visits. Clinical services should focus on periodic syphilis and HIV screening, personalized risk-reduction counselling including condom negotiation skills. Marital and regular partners of MSWs are also at high risk for HIV infection. Hence, effective mechanisms for partner management need to be established. Given the high HIV prevalence and vulnerability of MSWs, studies on the feasibility and acceptability of the newer bio-medical interventions such as pre-exposure prophylaxis and treatment as prevention could be considered.

## Competing interests

The authors declare that they have no competing interests.

## Authors’ contributions

PN conceptualized, carried out literature review, analysed the data and wrote the manuscript. GM and AD participated in the design of the manuscript and guided the analysis and manuscript development. AD restructured the manuscript. GD carried out the laboratory investigations and reviewed the manuscript. AR, AD, PP and RG conceived the study and reviewed the manuscript. All authors read and approved the final manuscript.

## Pre-publication history

The pre-publication history for this paper can be accessed here:

http://www.biomedcentral.com/1471-2458/13/1059/prepub
